# Homologs of the *Brugia malayi *diagnostic antigen *Bm*R1 are present in other filarial parasites but induce different humoral immune responses

**DOI:** 10.1186/1475-2883-3-10

**Published:** 2004-12-31

**Authors:** Rahmah Noordin, Ros Azeana Abdul Aziz, Balachandran Ravindran

**Affiliations:** 1 Institute for Research in Molecular Medicine and School of Medical Sciences, Universiti Sains Malaysia, Malaysia; 2 Division of Immunology, Regional Medical Research Centre, Indian Council of Medical Research, Bhubaneswar, India

## Abstract

**Background:**

The recombinant antigen *Bm*R1 has been extensively employed in both ELISA and immunochromatographic rapid dipstick (Brugia Rapid) formats for the specific and sensitive detection of IgG4 antibodies against the lymphatic filarial parasites *Brugia malayi *and *Brugia timori*. In sera of individuals infected with *Wuchereria bancrofti *the IgG4 reactivity to *Bm*R1 is variable, and cross-reactivity of sera from individuals infected with *Onchocerca volvulus *or *Loa loa *was observed only in single cases. In order to characterize the homologs of the *Bm*R1 antigen in *W. bancrofti *(Wb-*BmR1*), *O. volvulus *(Ov-*Bm*R1) and *L. loa *(Ll-*Bm*R1) the cDNA sequences were identified, the protein expressed and the antibody reactivity of patients' sera was studied.

**Methods:**

PCR methodology was used to identify the cDNA sequences from cDNA libraries and/or genomic DNA of *W. bancrofti*, *O. volvulus *and *L. loa*. The clones obtained were sequenced and compared to the cDNA sequence of *Bm*R1. Ov-*Bm*R1 and Ll-*Bm*R1 were expressed in *E. coli *and tested using an IgG4-ELISA with 262 serum samples from individuals with or without *B. malayi*, *W. bancrofti*, *O*. *volvulus *and *L. loa *infections or various other parasitic infections. *Bm*R1, Ov-*Bm*R1 and Ll-*Bm*R1 were also tested for reactivity with the other three IgG subclasses in patients' sera.

**Results:**

Wb-*Bm*R1 was found to be identical to *Bm*R1. Ov-*Bm*R1 and Ll-*Bm*R1 were found to be identical to each other and share 99.7% homology with *Bm*R1. The pattern of IgG4 recognition of all serum samples to *Bm*R1, Ov-*Bm*R1 and Ll-*Bm*R1 were identical. This included weak IgG4 reactivities demonstrated by *L. loa*- and *O. volvulus*-infected patients tested with Ov-*Bm*R1 and Ll-*Bm*R1 (or *Bm*R1). With respect to reactivity to other IgG subclasses, sera from *O. volvulus*- and *L. loa*-infected patients showed positive reactions (when tested with *Bm*R1, Ov-*Bm*R1 or Ll-BmR1 antigens) only with IgG1. No reactivity was observed with IgG2 or with IgG3. Similarly, ELISAs to detect reactivity to other anti-filarial IgG subclasses antibodies showed that sera from individuals infected with *B. malayi *or *W. bancrofti *(active infections as well as patients with chronic disease) were positive with *Bm*R1 only for IgG1 and were negative when tested with IgG2 and with IgG3 subclasses.

**Conclusions:**

This study demonstrates that homologs of the *Bm*R1 antigen are present in *W. bancrofti*, *O. volvulus *and *L. loa *and that these antigens are highly conserved. Recognition of this antigen by patients' sera is similar with regard to IgG1, IgG2 and IgG3, but different for IgG4 antibodies. We conclude that the *Bm*R1 antigen is suitable for detection of IgG4 antibodies in brugian filariasis. However, its homologs are not suitable for IgG4-based diagnosis of other filarial infections.

## Background

Lymphatic filariasis (LF) caused by *Brugia malayi *and *Brugia timori *is endemic in several Asian countries and infects approximately 13 million people. In May 2000, The Global Program for Elimination of Lymphatic Filariasis (GPELF ) was officially formed with the goal of eliminating the disease as a public health problem by the year 2020. To this end, sensitive and specific field-applicable diagnostic tools are required for mapping the distribution of the disease and monitoring the various phases of the program. Many areas endemic for LF are remote and have poor access to well-equipped laboratories, thus a rapid and field-applicable diagnostic test is important to ensure that it can be easily be performed by field workers and reliable, reproducible, results can be obtained. For bancroftian filariasis caused by *Wuchereria bancrofti*, the ICT antigen card test (Binax Inc., USA ) is widely used for this purpose. This test is based on the detection of a circulating adult worm antigen of *W. bancrofti*. Although this antigen is also present in *Brugia *[1], a reliable antigen detection test for human *B. malayi *infection is not available. Therefore, despite its inconvenience and insensitivity, routine diagnosis of brugian filariasis is made by light microscopy of night blood.

Although PCR assays are highly sensitive, these mainly detect individuals with circulating microfilariae (mf); and they are both time consuming and labour-intensive requiring well-equipped laboratory facilities.

Detection of anti-filarial IgG4 antibody provides a good alternative diagnostic tool for brugian filariasis, as this antibody subclass has been shown to be elevated in active infection and decline post-treatment [2-9]. Recombinant antigen-based antibody assays would be preferable over assays based on parasite extracts since the former allow for unlimited supply of well-defined antigens.

The *Bm*R1 recombinant antigen, expressed by gene pPROEXHT/*Bm17DIII *(GenBank accession no. AF225296), has been shown by us to be a highly specific and sensitive antigen for IgG4 assays to detect exposure to both *B. malayi *and *B. timori *infections. The antigen has been used in both ELISA and immunochromatographic rapid dipstick (Brugia Rapid) formats, and evaluation in various laboratories and field trials has revealed a sensitivity of 93%–100% in detecting microfilariaemic individuals [9-13]. Furthermore, in some endemic areas antibodies were also detected in amicrofilaraemic individuals, indicating the sensitivity of the assay in detecting sub-patent infections in brugian filariasis [10,13-15]. The *Bm*R1 antigen is highly specific (99%–100%) with respect to reactivity with sera from non-filarial infections [11,12]. The highest prevalence of cross-reacting antibodies in other filarial infections was found in *W. bancrofti*, followed by *Loa loa*, while only one sample of nine patients with *Dirofilaria *infection was found to be reactive. No cross-reactivity was exhibited in patients infected with *O. volvulus *or *Mansonella *[11,16].

Due to its diagnostic significance, it is therefore important to characterize the *BmR1 *antigen more closely. The varying degree of *Bm*R1 recognition in other filarial infection raises the question of whether the homologous antigen is also present in *W. bancrofti*, *L. loa *and *O. volvulus*. In the present study we have shown that *Bm*R1 antigen is highly conserved (99–100% amino acid identity), and that almost identical antigens are present in the other human filarial parasites of public health importance. Interestingly, however, the ability of the hosts to mount IgG4 response to *Bm*R1 homologs was found to be highly variable in some infections. In addition, the antibody responses of other IgG subclasses to *Bm*R1 and its homolog were also investigated.

## Materials & methods

### cDNA and genomic DNA

*W. bancrofti *microfilaria (mf), adult male and adult female cDNA libraries were obtained from the Filarial Genome Project Resource Centre (Smith College, Northampton, Massachusetts, USA genome@smith.edu). Genomic DNA of *W. bancrofti *mf were prepared from samples provided by Dr. B Ravindran, Division of Immunology, Regional Medical Research Centre, Indian Council of Medical Research, Bhubaneswar, India . The samples were comprised of two from individuals whose serum/blood samples were negative by the Brugia Rapid test and two from individuals who were positive by the Brugia Rapid test. *L. loa *L3 and *O. volvulus *mf cDNA libraries were kindly provided by Dr. P Fischer, Bernhard Nocht Institute for Tropical Medicine, Hamburg, Germany .

### PCR

To amplify the entire *Bm17DIII *gene sequence the following PCR primers were used: RNF (24 mer) 5' ATT ACT GAT TAG TAT TTT ATC GTT 3' and RNR (24 mer) 5'ATG ATA AAA ATG AAT GAG AAA TAT 3'. λ phage plaques were amplified and the DNA was extracted using a λ DNA extraction kit (Qiagen, Germany ). PCR was then performed in a thermocycler (Perkin Elmer, USA ) at the following conditions: 94°C, 5 mins.; 55°C, 5 mins.; 35 cycles for 94°C, 45 sec; 55°C, 45 sec & 72°C, 90 sec; 72°C, 10 mins.

Genomic DNA from *W. bancrofti *mf was prepared using Genispin Tissue DNA Kit (BioSynTech, Malaysia ). PCR amplifications using the above primers were then performed using the following thermocycler conditions: 94°C, 5 mins; 35 cycles for 94°C, 1 min; 55°C, 1 min & 72°C, 1 min; 72°C, 10 mins.

### TOPO cloning and DNA sequence analysis

For sequence analysis of the gene homologs in *W. bancrofti*, *O. volvulus *and *L. loa*, the PCR products were cloned into TOPO-TA vector (Invitrogen, USA ), then transformed into *E. coli *TOP10 host (Invitrogen, USA ). The recombinant plasmids were then amplified, purified using QIAprep^® ^Spin Miniprep Kit (Qiagen, Germany ), and subsequently sent for sequencing (ACGT Inc, USA ). The results of the DNA sequences were analyzed using vector NTI software (Invitrogen, USA ).

### Subcloning, expression and purification of *Ov17DIII/Ll17DIII*

The *Bm17DIII *gene homologs in *O. volvulus *and *L. loa *were subcloned into a bacterial expression vector, pPROEX-HT which contain 6-His tag (Life Technologies, USA ), then transformed into *E. coli *TOP 10 host cells.

The recombinant bacteria were cultured in Terrific broth and placed in a shaker incubator at 37°C until the optical density reached 0.5. The culture was then induced with 1 mM IPTG (isopropyl β-D-thiogalactopyranoside) for 3 hrs at 30°C. The bacterial pellet was reconstituted with lysis buffer containing 50 mM Tris HCl (pH 8.5), 5 mM 2-mercaptoethanol and a cocktail of protease inhibitors (Roche Diagnostics, Germany ). The suspension was sonicated at 200 W for 10 minutes, followed by centrifugation at 12 000 g for 30 minutes. The resulting supernatant was purified using Ni-NTA resin (Qiagen, Germany ) and buffers containing imidazole. The protein-containing fractions were then pooled.

### ELISA

The methodology employed was as previously reported [9]. Briefly, microtiter wells (Nunc, USA ) were coated with 100 μl of either *Bm*R1 (20 μg/ml) or the homologous recombinant antigens (5, 10 or 20 μg/ml) in NaHCO_3 _buffer (pH 9.6). After a blocking step, serum samples (1:20 or 1:50 or 1:100) were incubated for 2 h, followed by 0.5 h incubation with the secondary antibody HRP conjugated to monoclonal anti-human IgG1 (1:6000), IgG2 (1:1000, 1:2000), IgG3 (1:1000, 1:2000) or IgG4 (1:4500) (CLB Sanquin Blood Supply Foundation, Netherlands ). Subsequently ABTS substrate (Roche Diagnostics, ) was added for 30 minutes before the optical densities (OD) were read at 410 nm with an ELISA spectrophotometer (Dynatech (now DYNEX Technologies), USA ).

Serum samples were from existing serum banks, collected according to the ethical requirements of each institution. The samples were as follows: *O. volvulus*, *L. loa*, *W. bancrofti*, *B. malayi *and other parasitic infections. In addition serum samples from endemic normals (healthy and Brugia Rapid negative individuals from endemic areas in Malaysia) and non-endemic normals (healthy blood donors from Malaysia) were also tested. The *O. volvulus *sera were from microfilaremic from western Uganda [17]. *L. loa *sera were from microfilaremic individuals from the clinical department of the Bernhard Nocht Institute for Tropical Medicine, Hamburg, Germany. *W. bancrofti *sera samples were from India; while sera from *B. malayi *infections, endemic normals, non-endemic normals and other parasitic infections were from Malaysia. Infections with other parasites comprised of patients from Malaysia:

• whose stool specimens were positive for parasite ova/larva (single or mixed infections with Ascaris *lumbricoides*, *Trichuris trichiura*, hookworm, *Strongyloides stercoralis*)

• with clinical presentation and serology consistent with toxocariasis and amoebiasis

• with *Gnathostoma spinigerum *isolated from the eye (one patient)

## Results

### Identification of the *Bm*R1 homolog in *W. bancrofti*, *O. volvulus *and *L. loa*

In order to explain the pattern of antigen recognition in patients with other filarial infection we identified the homologs of the *Bm*R1 antigen in *W. bancrofti *(Wb-*Bm*R1), *O. volvulus *(Ov-*Bm*R1) and *L. loa *(LI-*Bm*R1). PCR of *W. bancrofti *cDNA libraries and *W. bancrofti *genomic DNA (from all 4 mf samples) produced a single band of 618 bp. PCR products were eluted from band 618 and cloned into TOPO vector. A total of 12 recombinant clones from six TOPO reactions (2 from mf cDNA, 1 from adult female cDNA, 1 from adult male cDNA and 2 from mf genomic DNA) were sequenced. A total of 31 DNA sequencing reactions were analyzed and all obtained sequences were identical. Comparison of the obtained nucleotide sequence showed that it was identical to cDNA sequence of *Bm*R1, irrespective whether the template DNA came from cDNA libraries, or microfilaria originated from individuals positive or negative for Brugia Rapid.

**Figure 1:**
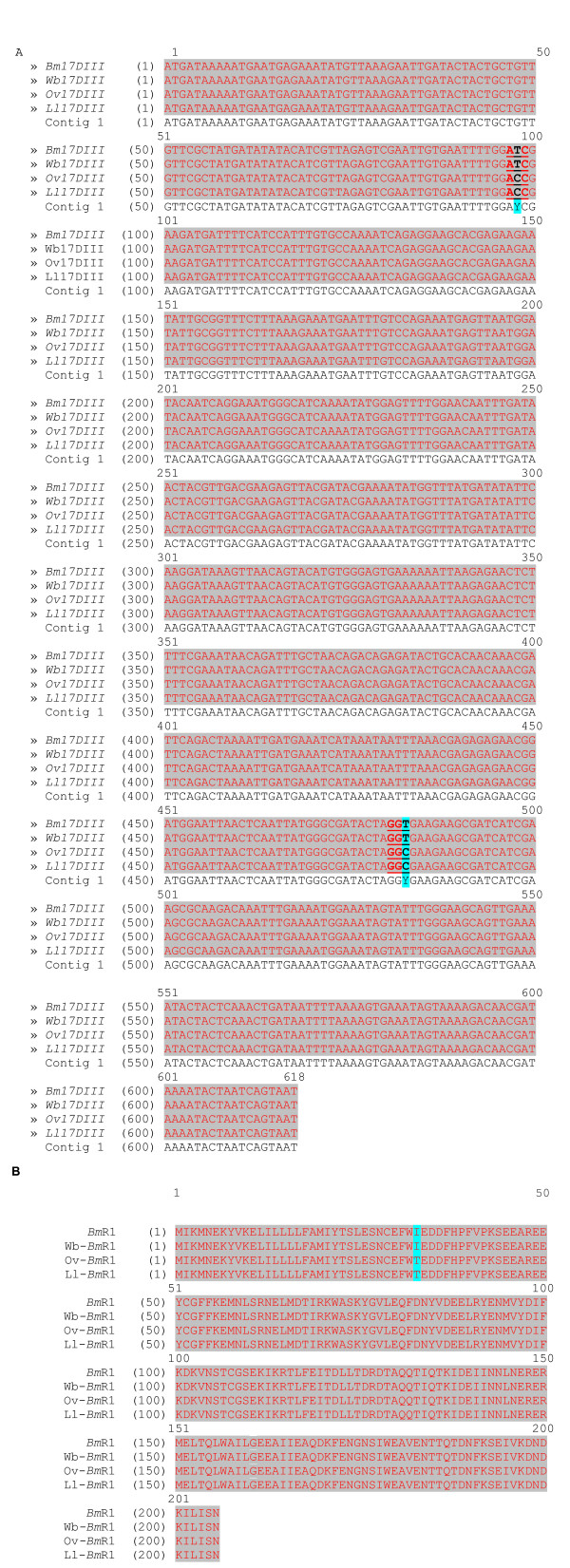
**a) Nucleotide sequence of *Bm17DIII *and its homologs in *W. bancrofti*, *O. volvulus and L. loa***. Top sequence data shows DNA sequences of *Bm17DIII *and its homologs *in W. bancrofti*, *O. volvulus *and *L. loa*. **b) Amino acid sequence of *Bm*R1 and its homologs in *W. bancrofti*, *O. volvulus and L. loa. ***Bottom sequence data shows amino acid sequence of *Bm*R1 and its homologs in *W. bancrofti *(Wb-*Bm*R1), *O. volulus *(Ov-*Bm*R1) and *L. loa *(Ll-*Bm*R1). Comparison between *Bm17DIII *DNA sequence and its DNA homologs in *O. volvulus *and *L. loa *showed only two bases difference at 98 and 483. Bm*R1 *homologs of the amino acid sequence was identical with *W. bancrofti*. However, with *O. volvulus *and *L. loa *a difference occurred only at one amino acid coded by bases 97–99 i.e. a change from Ile (ATC) to Thr (ACC).

For identification of the cDNA of the Ov-*Bm*R1 and Ll-*Bm*R1, a total of 5 and 3 recombinant clones were sequenced, respectively (comprising a total of 20 reactions). The Ov-*Bm*R1 and Ll-*Bm*R1 were 100% identical to each other, and only two base pairs were different from *Bm*R1 and Wb-*Bm*R1 i.e. at 97 bp and 483 bp. When the amino acid sequences were compared only one amino acid difference was observed: the uncharged polar isoleucine at position 33 was substituted by a neutral threonine (Figure 1).

### Antibody reactivity to *Bm*R1, Ov-*Bm*R1 and Ll-*Bm*R1 recombinant antigens

For IgG4-ELISA, serum samples that demonstrated average optical density (OD) readings of ≥0.300 were considered to be positive [9]. The comparison of IgG4 reactivites with *Bm*R1 and its homologs (Ov-*Bm*R1 and Ll-*Bm*R1) using a panel of 201 sera samples from individuals with various parasitic infections and 61 healthy controls (29 endemic normals and 32 non-endemic normals) indicated that the exchange of one amino acid had no influence on the reactivity of IgG4 antibodies. The IgG4-ELISA results showed all recombinant antigens were identical in reactivity with the various categories of sera (Table 1).

**Table 1:** Comparison among IgG4 reactivities of *Bm*R1, Ov-*Bm*R1 and Ll-*Bm*R1 using a panel of serum samples from patients with various parasitic infections and healthy controls (endemic and non-endemic normals). *Bm*R1 is the antigen expressed by *Bm17DIII *DNA sequence; while Ov-*Bm*R1 and Ll-*Bm*R1 are the antigens expressed by the homologs of *Bm17DIII *DNA sequence in *O. volvulus *and *L. loa *respectively.

**Serum type**	**No**	**Positive by BmR1 (%)**	**Positive by Ov-*Bm*R1 and Ll-*Bm*R1 (%)**
*O. volvulus*, mf positive	70	1 (1.43)	1 (1.43)
*L. loa*, mf positive	14	6 (42.86)	6 (42.86)
*W. bancrofti*, mf positive	33	8 (24.24)	8 (24.24)
*B. malayi*, mf positive	28	28 (100)	28 (100)
*Trichuris trichiura*	8	0	0
*Ascaris lumbricoides*	8	0	0
Mixed infection with *T. trichuris*, *A. lumbricoides *and hookworm	8	0	0
*Entamoeba histolytica *(invasive)	11	0	0
*Toxocara*	14	0	0
*Gnathostoma spinigerum*	1	0	0
*Strongyloides stercoralis*	6	0	0
Endemic normals *(healthy controls)*	29	0	0
Non-endemic normals *(healthy controls)*	32	0	0
**TOTAL**	**262**		

Reactivities of *Bm*R1 and its homologs (Ov-*Bm*R1 and Ll-*Bm*R1) with serum antibodies of the other three IgG subclasses (IgG1, IgG2 and IgG3) using samples from *O. volvulus *and *L. loa *infected individuals showed positive reactions with only IgG1. Most IgG1 positive samples had an OD >1.000. Similarly, the reactivities of anti-filarial IgG1, IgG2 and IgG3 antibody subclasses with *Bm*R1 on serum samples from active and chronic cases of *W. bancrofti *and *B. malayi *showed positive reactions only with IgG1. It is also noted that sera from non-endemic normals and soil-transmitted infections also showed similar reactivities i.e. IgG1 positive and IgG2- & IgG3-negative (Table 2).

**Table 2:** Results of ELISAs to detect IgG1, IgG2 and IgG3 anti-filarial antibodies in serum samples from patients with various helminthiasis and healthy controls (non-endemic normals) using *Bm*R1, Ov-*Bm*R1 and Ll-*Bm*R1. All antigens (tested separately) demonstrated identical results with all serum samples.

**Type of serum sample**	**Number of positive results out of number of samples tested**		
	
	IgG1-ELISA	IgG2-ELISA	IgG3-ELISA
*O. volvulus *mf+	47/47	0/21	0/21
*L. loa *mf+	14/14	0/14	0/14
*W. bancrofti mf*+	6/6	0/6	0/6
*W. bancrofti *chronic	6/6	0/6	0/6
*B. malayi *mf+	10/10	0/10	0/10
*B. malayi *chronic	14/14	0/14	0/14
Soil-transmitted helminth infections	10/10	0/10	0/10
Non-endemic normals (healthy controls)	10/10	0/10	0/10

## Discussion

*Bm*R1, a recombinant *B. malayi *antigen of ~30 kDa expressed by *Bm17DIII *DNA coding sequence (cds), has been consistently shown to be a sensitive and specific antigen for the immunodiagnosis of brugian filariasis in studies employing either ELISA or immunochromatographic rapid test (Brugia Rapid) formats [9,11-13,15]. When compared with the DNA sequences in GenBank, *Bm17DIII *cds exhibited 94% homology with the reported EST sequence derived from *B. malayi *microfilaria cDNA (GenBank AW244981). Southern blot hybridization assays performed on cDNA libraries of L3, L4, mf, adult male and adult female *B. malayi *showed that it is present in all of the above stages (Rahmah *et al*., unpublished data). Bands of the correct molecular weight were observed in a Western blot of *B. malayi *mf, adult male and adult female soluble antigens probed with monopurified antibody to *Bm*R1 (Rahmah et al., unpublished data).

Multicenter evaluations performed with Brugia Rapid showed variable reactivity of *Bm*R1 to sera of *W. bancrofti*-infected patients. Reactivity to sera from Chennai, India was 54.5% (12/22); from Indonesia was 70% (14/20) and from the Cook Islands was 90% (9/10) [12,15]. The wide variation in the reactivity of the assay in Bancroftian filariasis in the above three geographical areas prompted us to undertake the current investigation. The present study has shown that the homolog in *W. bancrofti *is identical to the cDNA of *Bm*R1 – irrespective of the source of the parasites – whether the mf were isolated from the individuals whose sera showed positive or negative reactivity with the Brugia Rapid test. Thus the observed differences in the reactivity of *Bm*R1 antigen with *W. bancrofti *sera collected from different geographical regions does not appear to be due to genotypic variability between different isolates of mf. Further studies are currently underway to determine if the variability in the expression of the gene could account for the variability in the Brugia Rapid results with serum samples collected from *W. bancrofti *infected individuals.

PCR experiments were performed on the *W. bancrofti *genomic DNA samples to obtain an amplicon with a size greater than 618 bp (since an intron is expected to be present in genomic material). However, only one prominent band of 618 bp was obtained (very occasionally a faint band of >1 kb was observed which was shown later to be due to unspecific amplification). PCR on *W. bancrofti *genomic DNA to amplify the intron sequence (using primers based on the *Bm17DIII *intron) produced a sequence that shared ~75% homology to the intron of *Bm17DIII*. This is believed to be an amplification on another part of *W. bancrofti *genome, since PCR using a pair of internal primers that flank the possible intron site produced a PCR product of ~300 bp (a size that is expected if there was no intron). Conversely, amplification of *B. malayi *genomic material produced two kinds of amplicons: 618 bp and 1010 bp. The latter was comprised of an intron (393 bp) and two flanking exons (237 bp and 381 bp), the sequences of which were consistent with *B. malayi *data at TIGR website . Thus at Universiti Sains Malaysia, genomic DNA of *Wb17DIII *was found to be intronless, whereas genomic DNA of *Bm*17DIII was shown to have two variants (i.e one with and one without an intron). These results, though seemingly controversial, were a result of exhaustive efforts with appropriate PCR controls. Data from other laboratories will hopefully confirm these results.

Anti-*Bm*R1 IgG4 was detected in 84.6% (44/52) of *L. loa *sera but generally not detected in *O. volvulus *serum samples [11,16]. Ov-*Bm*R1 and Ll-*Bm*R1 were identical to each other and 99.7% similar to *Bm*R1 (and to Wb-*Bm*R1) on the nucleotide level (Figure 1). Ov-*Bm*R1 and Ll-*Bm*R1 were found to display identical reactivity compared to *Bm*R1 when tested with IgG4-ELISA on a panel of serum samples (Tables 1 &2). Therefore, the difference of one amino acid between *Bm*R1 and its homologs (Ov-*Bm*R1 and Ll-*Bm*R1) did not alter their antigenicity. It is interesting to note that although IgG4 has been shown to be elevated in onchocerciasis with assays using other recombinant antigens [18,19], the IgG4 reactivity to *Bm*R1 or Ov-*Bm*R1 in *O. volvulus *was generally negative. One possible explanation for this is that adult worms mostly express Ov-BmR1 and the immune response to *O. volvulus *is predominantly due to mf [20]. This may explain the very poor IgG4 response to *Bm*R1 and Ov-*Bm*R1. It is possible that the uptake of antigen from lymphatic filariae by antigen presenting cells is significantly different compared to *O. volvulus *(where adult worms and mf reside either in sub-dermal nodules or in the skin).

The *Bm*R1, Ov-*Bm*R1 and Ll-*Bm*R1 recombinant antigens were also used to determine if IgG1, IgG2 or IgG3 antibodies in *O. volvulus *and *L. loa *serum samples were reactive with the recombinant proteins. In addition, the three IgG subclasses were also tested with *Bm*R1 on assays using sera collected from patients with *B. malayi *and *W. bancrofti *infections. In all cases only anti-filarial IgG1 was reactive, while anti-filarial IgG2 and IgG3 assays were consistently negative. It is important to note that IgG1 antibodies to *Bm*R1 and its homologs are unspecific and without any diagnostic value. The *Bm*R1 antigen obviously contains widespread epitopes that are recognized by IgG1 antibodies.

Thus based on the current study, *Bm*R1 and its homologs in *W. bancrofti*, *O. volvulus *and *L. loa *induce IgG antibody responses restricted to IgG1 and IgG 4 subclasses only. Unlike the anti-filarial IgG4 response in *B. malayi *infection, the IgG4 response to *Bm*R1 in *W. bancrofti *and *L. loa *was not consistently detected in all infected individuals, indicating that this recombinant antigen will not be of much use in the diagnosis of these two filarial infections. Although IgG1 response to *Bm*R1 was observed in all the filarial infections tested, it lacks specificity since it was also positive when tested with serum samples from normal individuals and with those infected with other parasites.

## Conclusions

The study demonstrates the presence of identical and almost identical homologs of the diagnostic *Bm*R1 antigen in other filarial parasites. However, they do not seem to induce consistent antibody responses in all infected subjects. Thus the immunogenicity of *Bm*R1 in brugian filariasis appears to be clearly different from that of bancroftian filariasis, onchocerciasis and loiasis.

## Competing interests

The author(s) declare that they have no competing interests.

## Authors' contributions

RN – was the principle researcher, designed the study, supervised the experiments and result analysis, wrote the first draft of the manuscript.

RAAA – performed the experiments and participated in the analysis of the data.

BR – provided parasite materials, collected patients' sera, edited the paper.
